# Finite Element Modeling Application in Forensic Practice: A Periprosthetic Femoral Fracture Case Study

**DOI:** 10.3389/fbioe.2020.00619

**Published:** 2020-06-23

**Authors:** Mara Terzini, Alessandra Aldieri, Stefania Nurisso, Giuseppe De Nisco, Cristina Bignardi

**Affiliations:** ^1^Department of Mechanical and Aerospace Engineering, Politecnico di Torino, Turin, Italy; ^2^PolitoBIOMed Lab, Politecnico di Torino, Turin, Italy

**Keywords:** periprosthetic fractures, FE analysis, fall loads, orthopedic plates, fractures types

## Abstract

The incidence of periprosthetic fractures has rapidly increased in the last two decades and has been the cause of a large number of revision surgeries and permanent physical disability for many patients, as well as a significant socioeconomic burden for many nations. This research deals with a periprosthetic femur fracture real event, occurred following a total hip arthroplasty and treated with one of the most widespread internal fixation methods: the implant of a periprosthetic femur plate system. A Finite Element analysis was performed to investigate the implanted femur plate break after a short follow-up and to understand the plate break causes. Such events are currently object of forensic debate as more and more often hospitals, surgeons, and medical device manufacturers are denounced by patients to whom similar events occur. In this work, different load situations acting on the femur during daily and incidental activities were simulated, in order to validate the correct behavior of the plate, according to the intended use recommended by the manufacturer. The analysis demonstrates that the plate failure can occur in situations of unconventional loading such as that caused by stumbling and in presence of incomplete bone healing.

## Introduction

Periprosthetic fractures (PF) are bone accidents associated with an orthopedic implant, whether a replacement device or an internal fixation device. In the last 20 years, due to the growth in the percentage of elderly, there was an increase in the implanted hip prostheses and, consequently, in the rate of total hip arthroplasty revisions. The most common post-operative PF localization is the femur, with a higher incidence associated with total hip arthroplasty (THA) with respect those related to total knee arthroplasty (TKA) (Schwarzkopf et al., 2013; Capone et al., 2017). The periprosthetic femoral fractures (PFF) can range from minor injuries, with a minimal effect on the patient's outcome, to being catastrophic and drastically reduce the patient's quality of life. Furthermore, the increase of the PF related to THA during the last two decades, has been a worldwide considerable economic burden for the national economies. A reference is given in a study conducted by Lyons et al. that analyses the mean cost of the treatment per patient, ranging from 14600.00 € to 27000.00 € (Lyons et al., 2018). The PFF can be classified into intraoperative or post-operative: intraoperative PFF occurs during the surgical procedure, while post-operative PFF occurs averagely in the first decade from the intervention and is more frequent in patients with prior total knee or revision total hip arthroplasty. The incidence of post-operative PFF, according to the actual available literature, range from 0.1 to 18% of the primary total hip arthroplasty interventions and from 4 to 11% of the hip replacement ones (Schwarzkopf et al., 2013; Capone et al., 2017).

The risk factors influencing the occurrence of PFF can be related to patient condition, surgical intervention type and quality, and to implant features. The PFF are often determined by the mechanical properties adjustment of the implant-surrounding bone stock and the preoperative mechanical quality of the bone structure itself. For this reason patient's advanced age, osteoporosis, rheumatoid arthritis and other pathologic bone conditions are significant risks factors for tardive PFF, as well as implant loosening, implantation technique and type of implant. Body mass index, according to the current state of the art, do not affect the probability of PFF (Singh et al., 2013). Approximately, in the 75% of the cases, these fractures are caused by a low energy trauma, such as a low energy fall from sitting or standing (Katz et al., 2014; Frenzel et al., 2015).

Many PFF classification systems has been proposed that generally provide information about fracture location, fracture pattern, implant stability, and potential for loosening. Of all the proposed classification techniques, the Vancouver one (Duncan and Masri, 1995) is the most widely applied. It includes information about fracture location, pattern and implant stability. It is reliable, simple and reproducible and permits to identify a treatment strategy basing on easily identifiable fracture features (Marsland and Mears, 2012; Schwarzkopf et al., 2013). It classifies the PFF associated with total hip replacement in three types, according to the fracture location: A (around trochanteric region), B (the bed supporting or adjacent to the implant is involved and represent ~80% of the cases), and C (the diaphyseal area distal respect to the bed of the implant is involved). The Vancouver classification includes some general indications about the recommended treatment for each fracture type. Despite this, many treatment options are described in literature and no single treatment for each type of fracture has shown to be the gold standard.

In detail, type C fractures, object of this study, is currently treated with open reduction and internal fixation (ORIF) techniques, with the eventual add of cortical strut allograft depending on the bone stock quality (Schwarzkopf et al., 2013). The ORIF strategy involves the implant of an external plate fixed upstream and downstream to the fracture site by means of screws, cables or other strategies, in order to promote bone reduction. In particular situations, it can be useful to implant an intramedullary device. The total surgical revision rate after PFF treatment is around 16.5% and, in almost all cases, failure occurs in the first year after surgery. Generally, failure reasons are plate loosening or breaking and infection for fracture treated with ORIF and stem subsidence, hip dislocation and infection for fracture treated with revision surgery. The mortality rate during the firsts 30 days after surgery is around 1.6% and the 1-year mortality rate is around 13.2%. After 1 year usually this value decrease with time. The highest mortality failure rate is associated with Vancouver type B fractures with bad bone stock quality (Füchtmeier et al., 2015).

The structural analysis of skeletal body elements and of biomechanical systems consisting of a bone element coupled to a prosthesis, an implant or a fracture synthesis device, can be performed both numerically and experimentally. There are indeed many examples of clinical problems which have moved from a qualitative assessment to a quantitative evaluation thanks to computational modeling (Vitale et al., 2008; Zanetti and Bignardi, 2013; Zanetti et al., 2017, 2018a; Aldieri et al., 2018b; Calì et al., 2018; Putzer et al., 2018; Terzini et al., 2018, 2019; Putame et al., 2019), to the application of classical experimental methods of structural analysis in the evaluation of the efficacy of procedures or surgical techniques (Bresciano et al., 2005; Menicucci et al., 2006; Zanetti and Audenino, 2010; Zanetti et al., 2012a, 2018b; Boero Baroncelli et al., 2013; Manzella et al., 2013, 2016; Bignardi et al., 2018), or to the evaluation of the mechanical characteristics of the materials used at different scales of investigation (Peluccio et al., 2007; Bignardi et al., 2010; Zanetti et al., 2012b; Terzini et al., 2016a,b; Aldieri et al., 2018a). Computational modeling, more than experimental one, finds natural application in forensic practice, as samples are often unavailable for experimental investigations. In this framework, it can be fundamental to determine whether an osteosynthesis medical device break is the consequence of an unconventional loading or of an error in designing or manufacturing the device. A methodology for assessing the causes of a given scenario is the use of Finite Element (FE) analysis. In the biomechanical evaluation of osteosynthesis medical devices, many applications can be found in the literature (Dubov et al., 2011; Noor et al., 2013), but to the authors' knowledge this is the first investigation aimed at evaluating the causes of a real failure event after a re-intervention following PFF. Such events are currently object of forensic debate as more and more often hospitals, surgeons, and medical device manufacturers are denounced by patients to whom similar events occur.

This study aims to quantitatively analyse, by means of a FE analysis, the possible causes of a real case of femoral plate break, implanted following a PFF. Different load conditions acting on the femur during daily and incidental activities were simulated, in order to validate the correct behavior of the plate, according to the intended use recommended by the manufacturer.

## Materials and Methods

### Case Description

A patient (age range 70–80 years) with a total right hip arthroplasty (THA), experienced, according to Vancouver classification, a type C periprosthetic fracture (Duncan and Masri, 1995) located in the distal femur; the fracture shape was spiroid and the bone stock was of good quality. Informed consent regarding the study of the clinical case and the publication of the radiographic images related to the follow-up is available. The reduction of the fracture was performed by means of a classical model of distal femoral plate. The surgeon chose to use a cerclage wire to secure to the bone the proximal region of the plate. All the screws are fixed in a bi-cortical way, except for the three proximal ones that are mono-cortical in order to avoid the contact with the prosthesis stem. An interfragmentary screw, crossing the fracture rime, was inserted. Diameters and lengths of plate screws are known. Materials constituting plate and plate screws are known while the material constituting the cerclage wire and the interfragmentary screw, as well as the model of the implanted prosthesis, are unknown.

**Figure 1:**
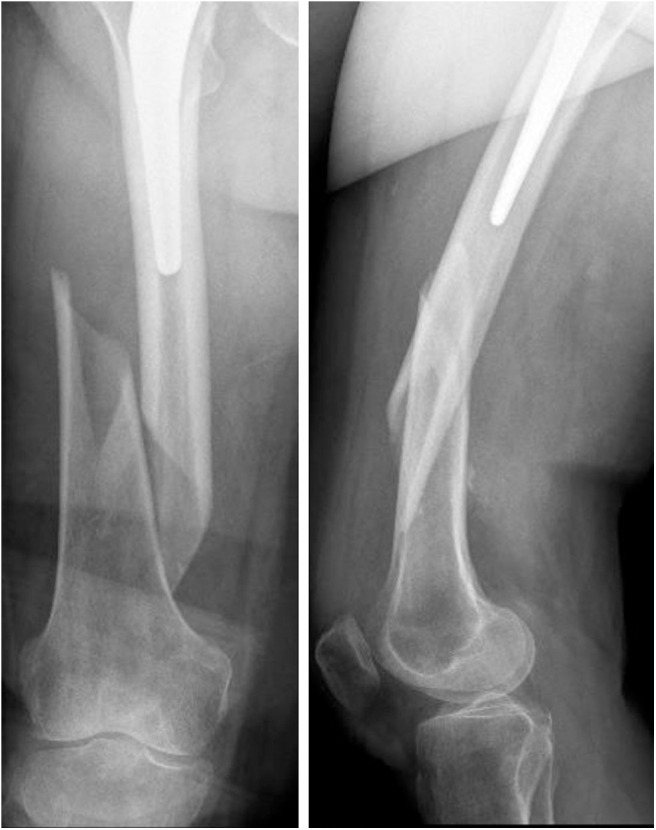
Anteroposterior **(Left)** and lateral **(Right)** x-ray images of the periprosthetic fracture.

**Figure 2:**
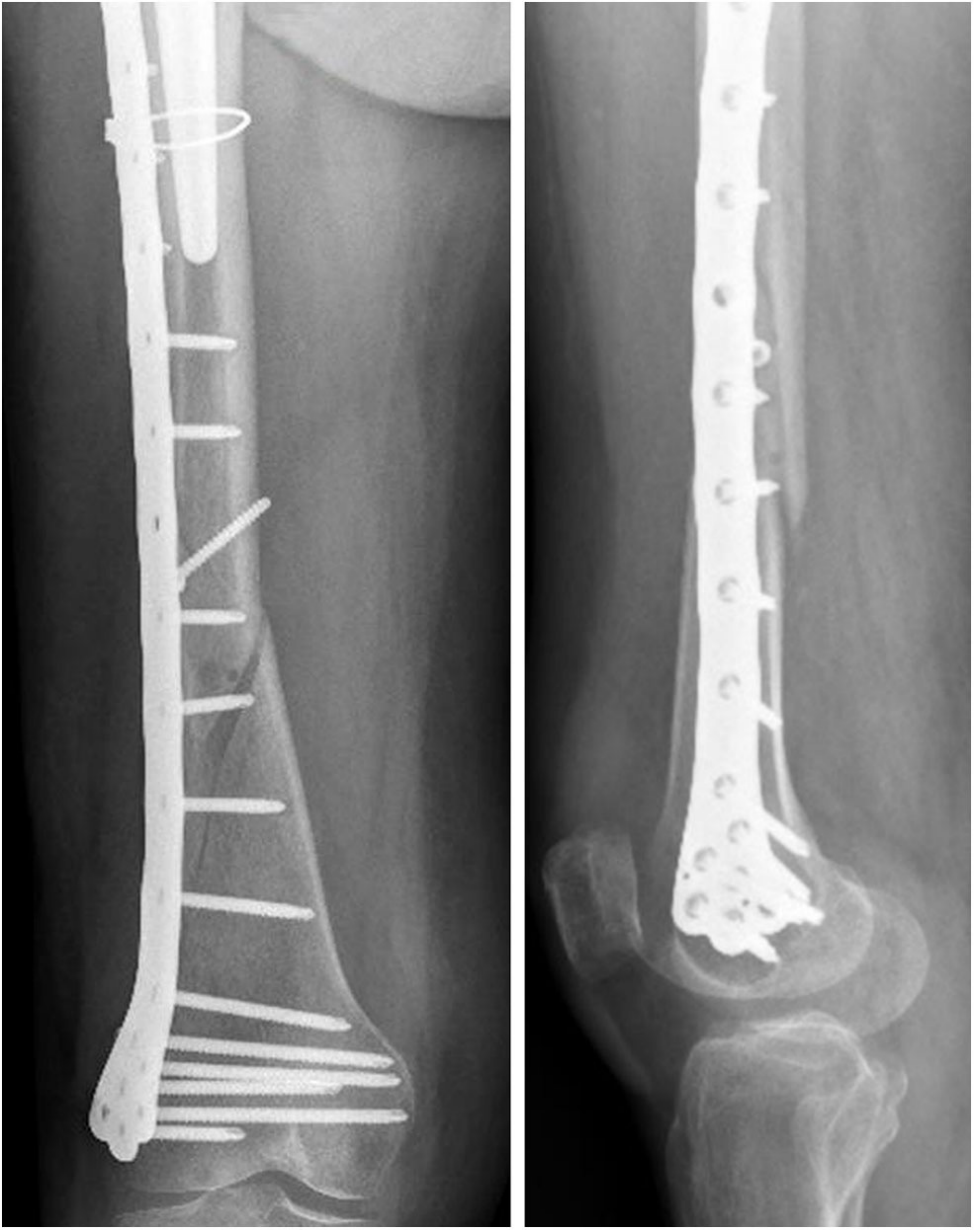
Anteroposterior **(Left)** and lateral **(Right)** x-ray images of the reduced periprosthetic fracture after the plate implant.

**Figure 3:**
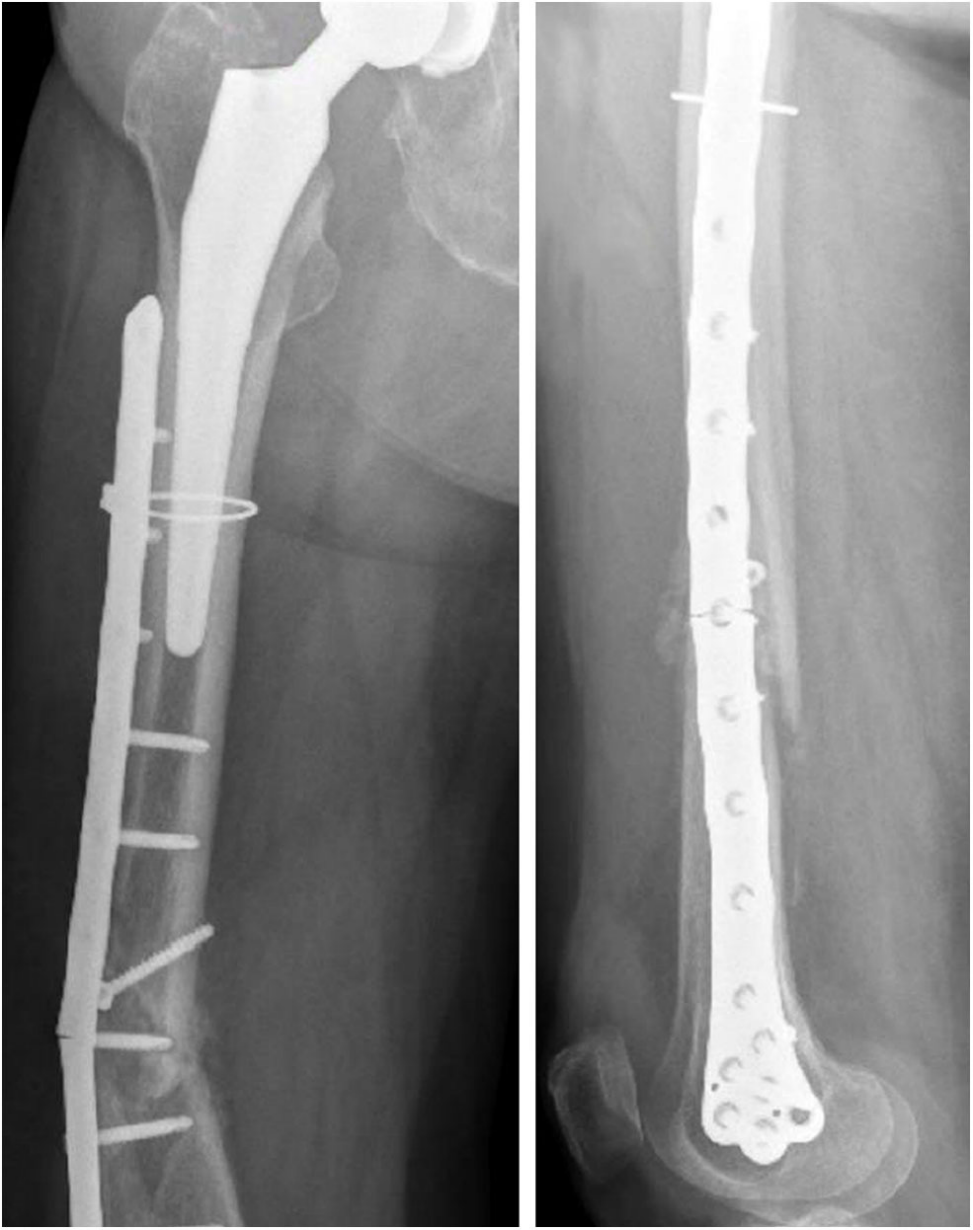
Anteroposterior **(Left)** and lateral **(Right)** x-ray images of the plate break.

The x-ray images of the femoral fracture are shown in Figure 1, while Figure 2 shows the x-ray images after the plate implant. After 3 months the plate suffered of catastrophic break in the area where the healing bone was present, accompanied by a further femoral fracture characterized by a profile similar to the previous one (Figure 3). The context and reason of the break are unknown.

The patient had no history of bone disease at the time of the accident, and the patient weight is unknown.

### Numerical Model

**Figure 4:**
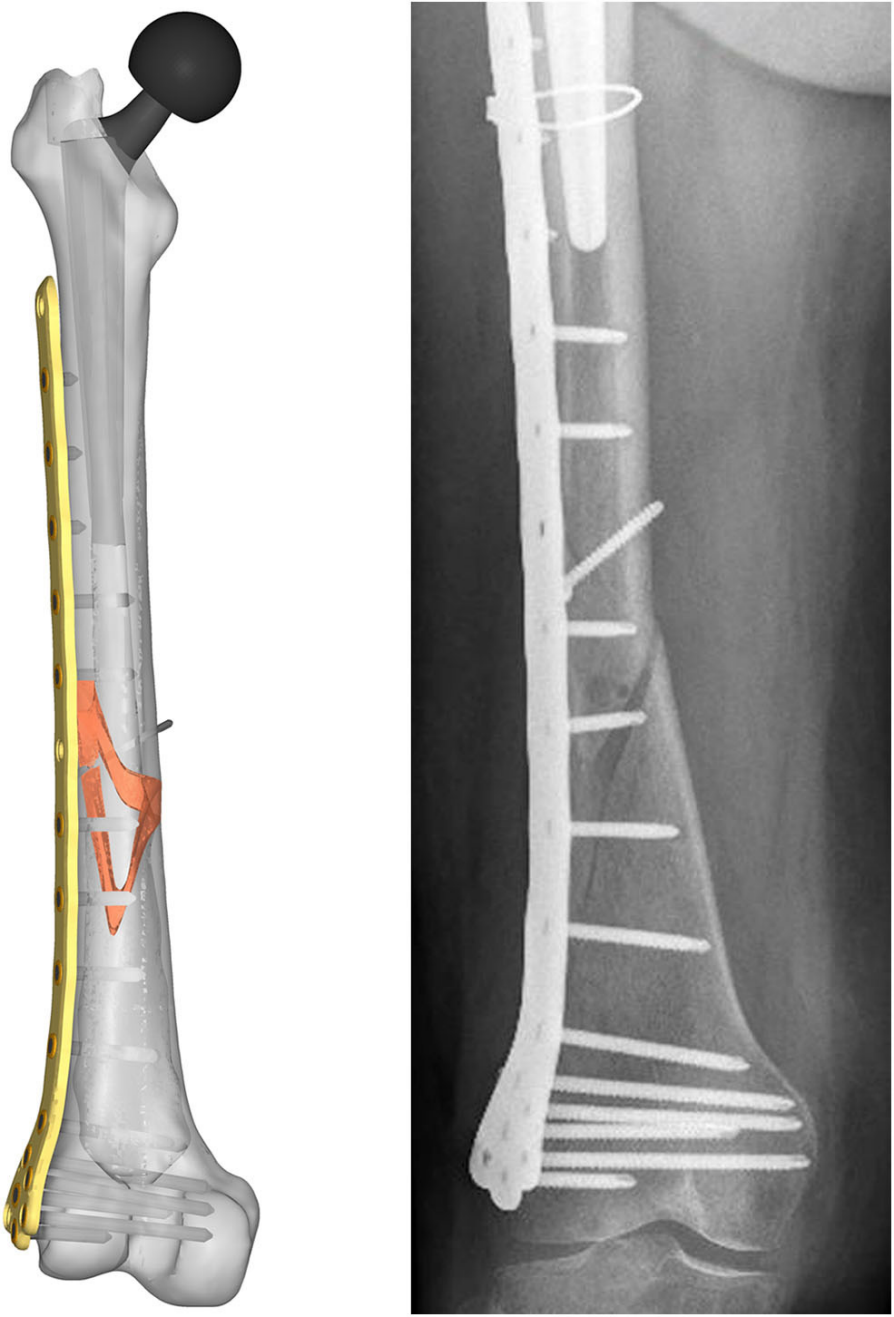
Resulting assembled geometrical model **(Left)** compared with the corresponding x-ray image **(Right)**.

**Figure 5:**
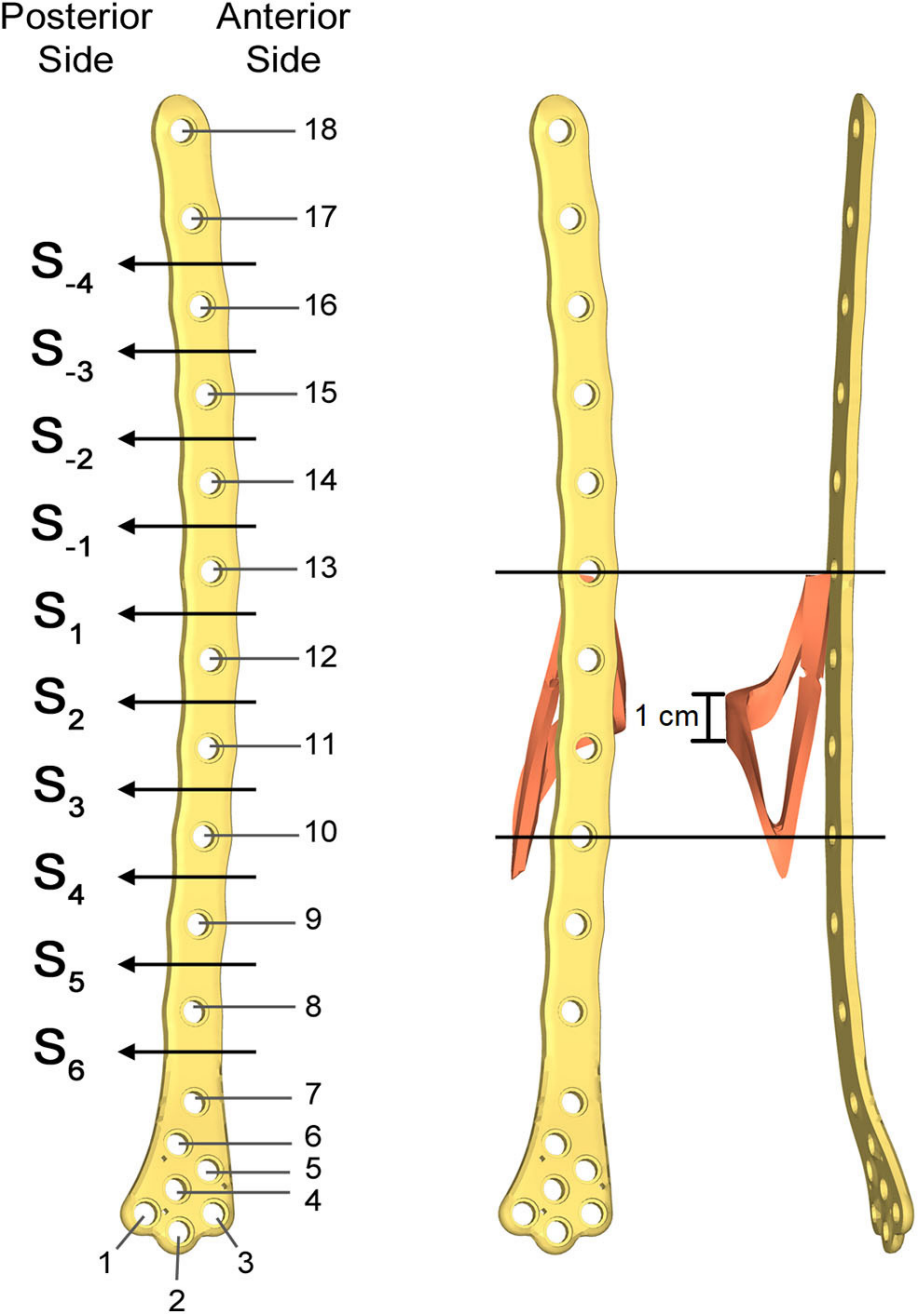
Numbering assigned to holes and zones of the plate **(Left)**; lateral **(Middle)**; and postero-anterior **(Right)** views of plate and bone callus volume. The longitudinal extension of the fracture is pointed out, as well as the fracture bone callus average thickness.

Aiming to analyse the structural behavior of the femur plate in the studied condition, and to identify the possible cause of its break, a 3D model replicating the configuration previously described was implemented in Solidworks (Dassault Systèmes, France) (Figure 4). A standard geometric model of an adult right femur containing only the cortical region geometry was used (Zanetti and Bignardi, 2009): the epiphyses were filled with cortical bone while the diaphysis was considered empty. These assumptions have negligible influence on the stresses on the plate, the main object of investigation of the present study (Papini et al., 2007). Using the software ImageJ (National Institutes of Health, USA) some reference measures were taken on the x-ray images in order to scale the standard femur model and adapt it to the real anatomical case. To recreate the fracture profile, reference points were identified on the x-ray images and reported on the bone surface, so that the fracture shape could be reproduced. The result is a fracture volume characterized by an average thickness of 1 cm (Figure 5).

As there was no available information on the implanted prosthesis, an available prosthetic stem model of appropriate size was chosen. Prosthesis neck length and head diameter were then modeled in Solidworks in order to respect the real joint center.

The femur plate implanted in the patient has 7 holes in the condylar area and 11 along the diaphyseal one, and plate CAD geometry was available. It was positioned relatively to the femur according to the x-ray images. For the sake of clarity, the 18 plate holes were numbered starting from the bottom and the zones of the plate between each pair of the 11 diaphyseal holes were numbered according to the distance from the bone fracture (– in the proximal direction and + in the distal direction), which is located at the 12th hole and extends downwards to the 10th hole (Figure 5). Note that the real plate break occurred at the 11th hole between S2 and S3 zones, as visible in Figure 3. Plate screws and bushings geometries were simplified to avoid the formation of too small and distracting elements during meshing, and therefore optimizing the computational time. In detail, too small filets and chamfers were removed from the bushings, and the threaded portion of the screws was replaced by a cylindrical geometry with a diameter equal to the screw core one. Screws length were singled out through measurements on the x-ray images. As the model of the implanted interfragmentary screw is unknown, a simplified screw geometric model was used, and the material was assumed to be the same of the plate screws. The presence of the cerclage wire, located in the area comprised between the last two proximal plate holes, was neglected as no screw failure in that region was detected.

The so assembled model, composed of the fractured femur, the prosthetic stem and head, the bushings, the plate screws, the interfragmentary screw, and the femur plate, was imported into the FE pre-processor software Hypermesh (Altair, USA). Table 1 lists the material properties assigned to each volume. A linear elastic behavior was assumed for each component, and cortical bone mechanical characteristics, considered homogeneous and isotropic, were chosen according to Morgan et al. (2018). Regarding the fracture bone callus, the mechanical properties are dependent on the specific situation. Bone tissue, in fact, has a remarkable ability for self-repair and regeneration after an injury, and is able to completely restore its mechanical function. The time needed for bone regeneration is patient specific, and early controlled by intrinsic genetic factors. Examining the research conducted by Manjubala et al. (2009) regarding the evaluation of the nanoindentation modulus of the callus formed during the bone healing, and considering the uncertainty regarding the actual patient bone reduction, different material properties were assigned to the fracture volume. These values were selected according to a linear proportionality factors of bone reduction starting from the maximum value of Young's modulus chosen for the healthy cortical bone (100% bone reduction factor). Considering that the fracture occurred after 3 months from the plate implant, an elastic modulus value equal to half of the healthy bone one was considered representative of the situation. Therefore, setting a 50% bone reduction factor as the maximum elastic modulus assignable to the fracture bone callus, decreasing percentages of 50, 20, 10, and 2% were selected (Table 2). The Poisson's ratio was considered to be equal to that of healthy bone in all the situations.

**Table 1:** Materials assigned to the model components and mechanical characteristics assumed (Srivastav, 2011).

**Component**	**Material**	**Young's modulus [MPa]**	**Poisson's ratio**
Prosthetic head, Prosthetic stem, Plate	Stainless steel 316LVM	210000.0	0.3
Femur	Cortical bone	18160.0	0.3
Fractured zone	Bone callus	Variable (see Table 2)	0.3
Plate bushings, Plate screws, Single screw	Ti6Al4V	110000.0	0.3

**Table 2:** Mechanical properties of bone callus for different assumed bone reduction factors.

**Bone reduction factor [%]**	**Young's modulus [MPa]**
50	9080.0
20	3632.0
10	1816.0
2	363.2

**Figure 6:**
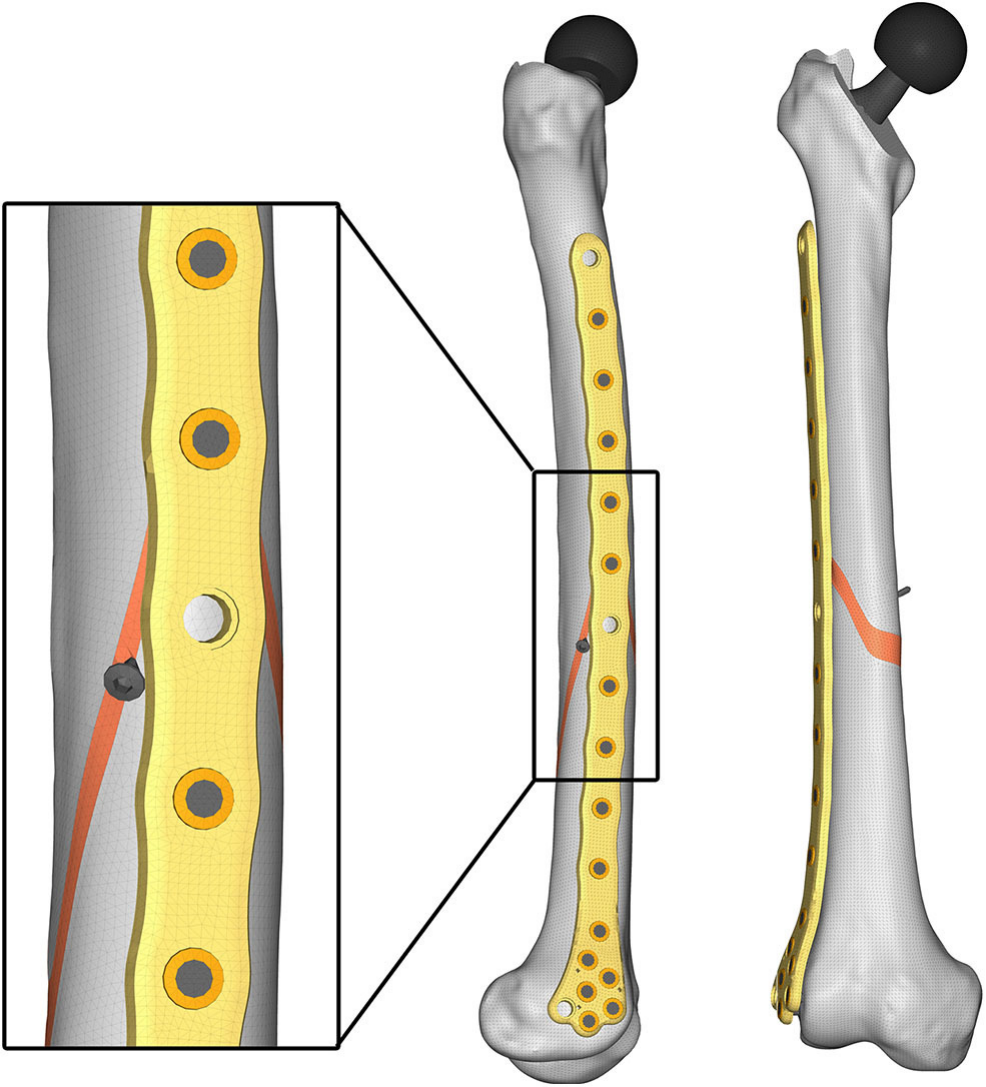
Lateral **(Left)** and anteroposterior **(Right)** views of the obtained mesh.

According to Viceconti et al. (1998), Polgar et al. (2001), and Ramos and Simões (2006), a tetrahedral mesh of 388652 elements with a 2 mm average edge length was created (Figure 6).

Given the aim of the research, in order to simplify the FE model, a congruent mesh between each component was preferred, thus generating a tie contact in the following component pairs: bone-screw, bone-prosthesis, screw-bushing, and bushing-plate. The bone-plate contact was neglected after verifying that no contact occurred between the two components under all load conditions.

**Figure 7:**
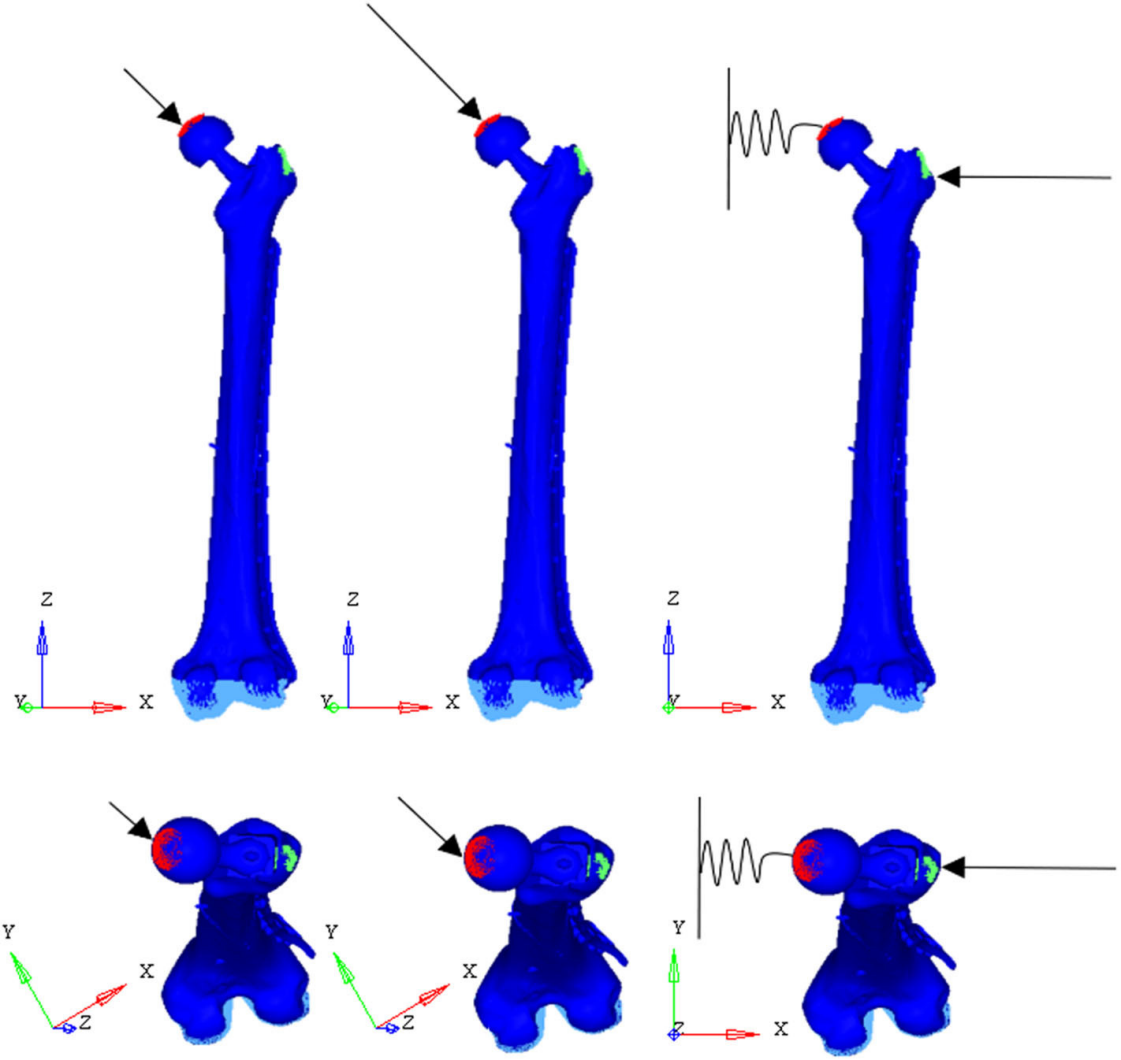
Applied load conditions: walking **(Left)** (Bergmann et al., 2001), stumbling **(Middle)** (Bergmann et al., 2004), and falling **(Right)** (Aldieri et al., 2018b).

To test the structural behavior of the plate, both daily and incidental load conditions were simulated. Indeed, the evaluation of stress and strain distributions in the plate under several conditions, enable to identify the load configuration able to overstress the plate beyond the breaking limit. In detail, three load configurations were implemented (Figure 7): (1) walking (according to Bergmann et al., 2001), (2) stumbling (according to Bergmann et al., 2004), and (3) lateral falling (according to Robinovitch and Hayes, 1991; Kheirollahi and Luo, 2015; Aldieri et al., 2018b). In the walking configuration and in the stumbling configuration the femur base was constrained along all the degrees of freedom, while in the falling configuration it was constrained with a hinge element that permits the only rotation along the y axis.

In the walking configuration the instant of the walking cycle in which the first of the two peaks of the contact force takes place was considered. In his work, Bergmann et al. (2001), obtained the contact force amplitude through experiments on four subjects with instrumented hip, and expressed it as a percentage of body weight. Here, the three force components were obtained based on a supposed 95 kg patient body mass. The same procedure was followed for the stumbling configuration.

In the falling configuration the prosthetic head was connected to the ground through a spring element with a 10,000 N/mm stiffness acting along the x-axis. The spring simulates the stiffness of the soft tissues that surround the hip and damp the impact that occurs during the fall. Also in this case, the body mass of the patient was supposed equal to 95 kg, while the patient height was supposed equal to 1.70 m. Patient mass and height were overestimated in order to verify the resistance of the plate in even worse conditions than the real ones.

Loads applied in the three simulated conditions are listed in Table 3. It should be noted that loads directions were implanted according with the examined literature, and the used coordinate systems are shown in Figure 7.

**Table 3:** Simulated load conditions: walking (Bergmann et al., 2001), stumbling (Bergmann et al., 2004), and lateral falling (Aldieri et al., 2018b).

**Load condition**	**Type of force**	**Force component [N]**		
		***X***	***Y***	***Z***
Walking	F_J_	−2141.7	494.5	−297.1
Stumbling	F_S_	−6479.1	1495.9	−898.8
Lateral Falling	F_LF_	0.0	−7688.6	0.0

*F_J_ is the Joint force applied on the prosthesis head, F_S_ is the force deriving from Stumbling applied on the prosthesis head and F_LF_ is the force deriving from the Lateral Falling applied on the great trochanter*.

A total of 12 models were simulated, combining four decreasing bone qualities of the fracture bone callus and three load configurations. The numerical analyses were performed by means of the Abaqus/Standard solver of Abaqus (Dassault Systèmes, France).

## Results

**Figure 8:**
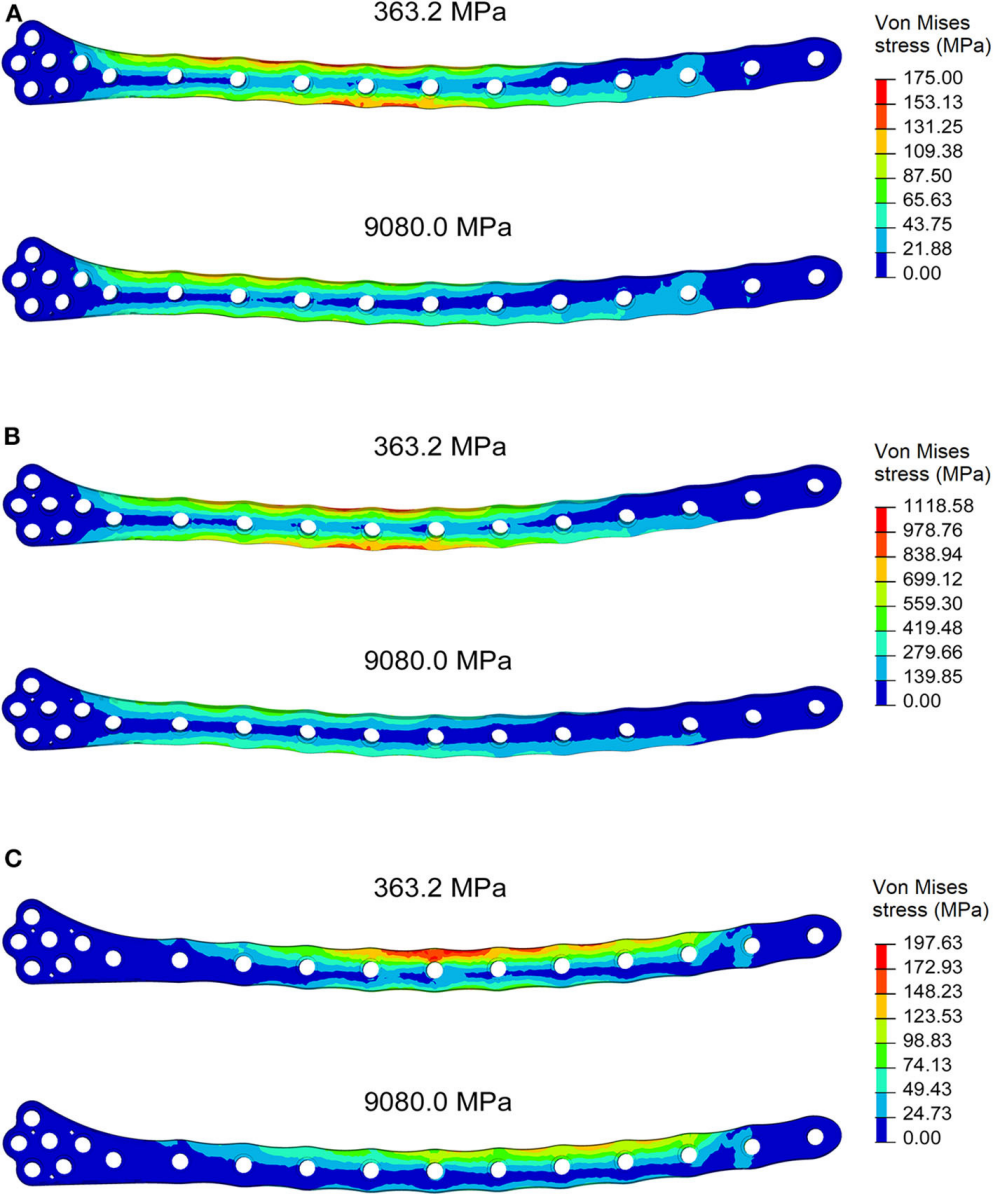
Von Mises stresses distribution (MPa) in the plate for the models with bone callus Young's modulus 363.2 and 9080.0 MPa resulting from **(A)** the walking condition, **(B)** the stumbling condition, and **(C)** the lateral falling condition.

**Figure 9:**
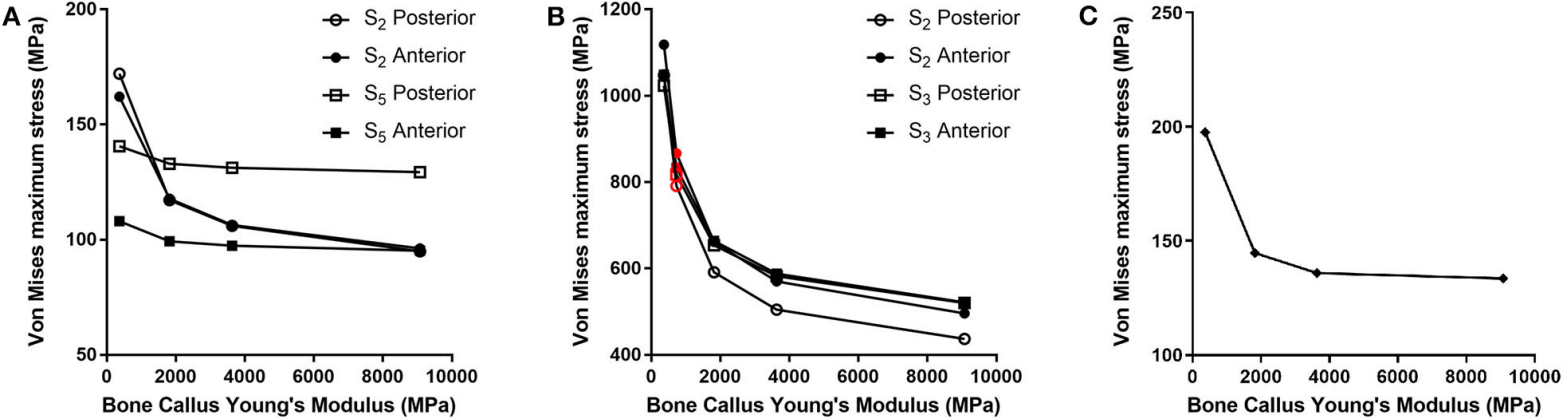
Von Mises maximum stresses (MPa) varying the bone callus Young's modulus resulting from **(A)** the walking load condition in the S2 and S5 zones (posterior and anterior areas); **(B)** the stumbling load condition, in the S2 and S3 zones (posterior and anterior areas). Results regarding a bone callus Young's modulus equal to 726.4 MPa are highlighted in red. **(C)** The lateral falling load condition.

For each of the load configurations considered, Von Mises stress in the femur plate and its peak value were analyzed. As regards the walking load configuration Von Mises stresses distribution within the plate varies according to the level of healing achieved. For lower Young's modulus values of the fracture bone callus, the lateral surfaces belonging to the S2 zone are more stressed. As the bone callus stiffness increases, the highest stresses move downwards, affecting the lateral surfaces of the S4 and S5 zones (Figure 8A), while the Von Mises stress values on the femur plate surface in the S2 zone reduce, with initially precipitous progression as visible in Figure 9A. In the S2 zone, the plate stress is distributed evenly between the anterior and the posterior portions, with a slight prevalence of the posterior area in the presence of lowest bone callus stiffness. Conversely, the S5 zone is characterized by a slightly decreasing trend both posteriorly and anteriorly, but with a clear predominance of the posterior values compared to the anterior ones.

In the less consolidated fracture situations, the applied load is transferred to the lower constraint through the femur plate, characterized by a Young's modulus three orders of magnitude higher than the fracture bone callus one. In the femur plate, indeed, the zones reaching higher Von Mises stresses are located between the 10th and the 13th holes, corresponding to the fracture region. As the level of healing increases, the higher stress region moves around S4 and S5 zones, where the bone cortical thickness is minimal. Here the bending moment given by the applied load is maximum, due to the greater distance from the point of application of the load.

However, in the walking load configuration, the maximum stress value reached in the most critical condition (i.e., the case of a bone callus Young's modulus equal to 363.2 MPa) is 248.0 MPa, which settles far below the plate yield point (about 700.0 MPa for stainless steel 316LVM; Srivastav, 2011).

The force acting on the femur during stumbling from standing position have the same direction as that during walking, but its module is amplified. In this case the zones where Von Mises stress is higher are S2 and S3, and in these zones the femur plate yield point is exceeded for lower values of the fracture bone callus Young's modulus (Figures 8B, 9B). This is interesting because, as previously stated, the real plate break occurred at the 11th hole between S2 and S3 zones. For this reason, an additional model was considered to better understand which level of bone healing would correspond to the plate break. Here, the bone callus Young's modulus was set equal to 726.4 MPa after few trials, corresponding to a bone reduction factor of 4%, and Von Mises stress results are represented in red in Figure 9B. The two stress distributions around the hole section where the actual break occurred show how the fracture bone callus Young's modulus influences the femur plate stress state (Figure 8B): indeed, as the fracture bone callus Young's modulus decreases, the higher stress zone moves closer to the 11th hole. The consequent yield causes the reduction of the plate section and the subsequent break. Being the most representative or the real break event, for the stumbling load condition a further model was considered in which a body mass equal to 75 kg, representative with high probability of the real situation, was addressed and the applied load was reduced accordingly. The obtained Von Mises stresses distribution was similar to the previous one, but as expected the stress peaks were lower and reached the yield stress value for a fracture bone callus Young's modulus equal to 300.0 MPa.

Also regarding the falling load configuration, the stress distribution within the femur plate varies according to the level of bone callus. The highest stresses were found, in any case, in the upper posterior side of the plate. These stresses grow and move downwards when the bone callus Young's modulus decreases (Figures 8C, 9C). For a bone callus with Young's modulus equal to 363.2 MPa, the maximum stress area covers the posterior side of the 12th hole, but the reached values settled below the plate yield stress value.

## Discussion

**Figure 10:**
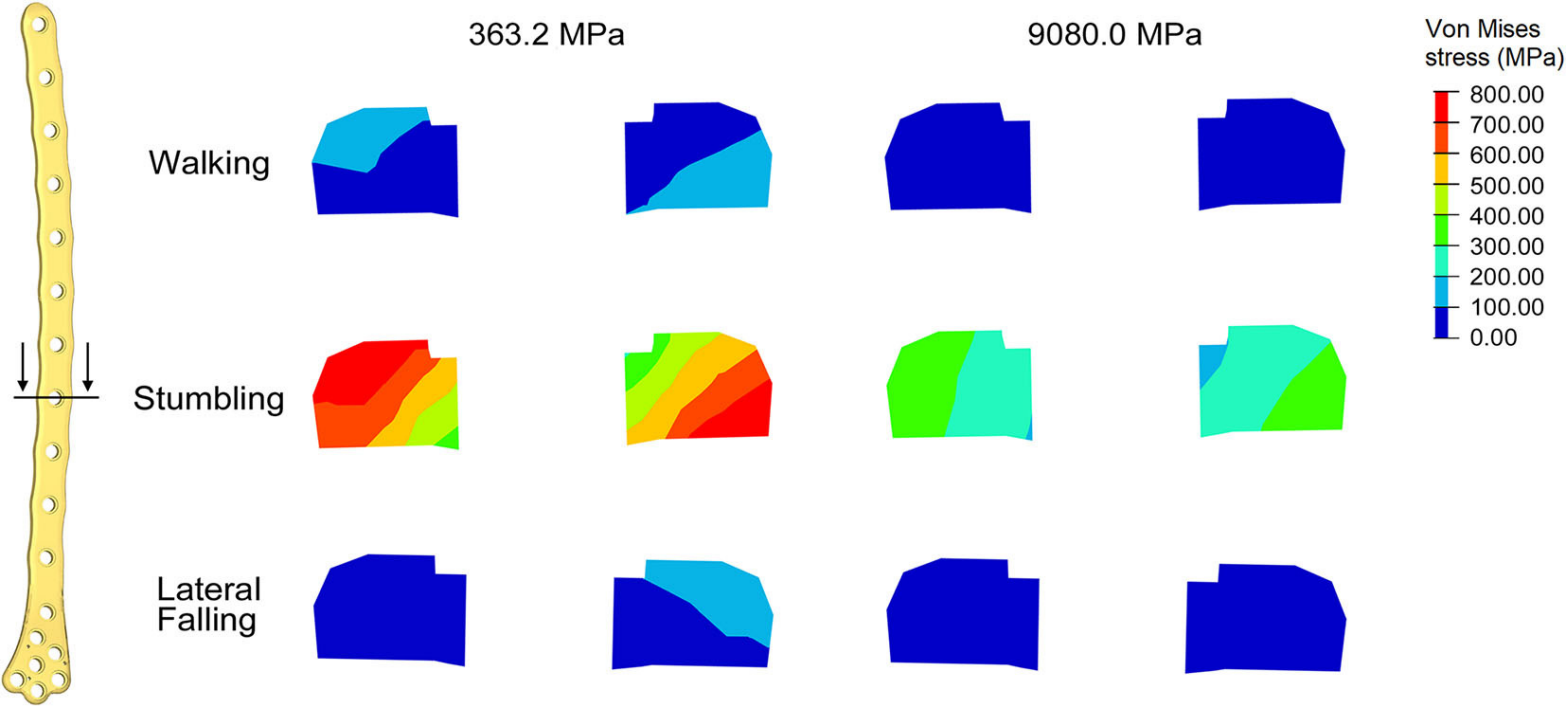
Von Mises stresses (MPa) in the cross-section corresponding to the 11th hole, were the plate break occurred. Results for the three loading condition are shown for the models with bone callus Young's modulus 363.2 and 9080.0 MPa.

**Figure 11:**
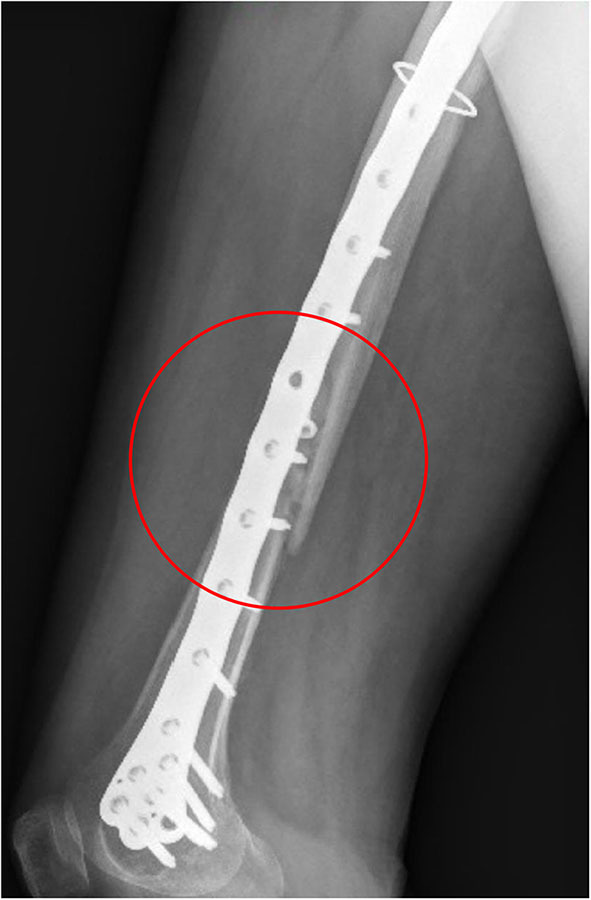
X-ray image taken after 2 months from the surgery. The non-consolidation of the bone fracture is pointed out.

Comparing the fracture bone callus Young's modulus and the locations of the stress peaks to which the plate would be subjected in the analyzed daily and incidental activities, it was concluded that, with a good probability, the break of the femur plate occurred due to the application of a load higher than 6,000 N and characterized by a predominant vertical component. In fact, this load condition would cause a concentration of the stress converging toward the 11th hole in correspondence of which the plate broke (Figure 10). These assumptions are valid for bone callus Young's modulus below ~1,500 MPa. This value is low compared to the level of bone healing reachable after 3 months from the surgery in optimal conditions. However, observing an x-ray acquired 2 months after the fixation (Figure 11), it seems that the fracture reduction was absent in the posterior area as the line that splits the bone fracture surfaces is clearly visible. Some of the possible causes could have been malalignment during surgery, incorrect distribution of stress that does not allow bone healing or pathologies that reduce the bone metabolism. The value of the bone callus Young's modulus that simulates at the best the real situation was however not known. Despite this lack, it was deduced that a combination of insufficient bone healing and unexpected high load applied to the prosthesis head with a predominant vertical component is the most probable cause of the plate break occurrence. In particular, the research highlighted how the femur plate implanted during surgery is able to support the loads deriving from daily activities, such as walking, even considering high body masses, as long as an adequate bone healing is granted.

## Conclusions

In this work a FE model reproducing a femur plate implant after a PFF event was implemented in order to investigate the actual load condition that may have led to the break of the femur plate. Since both the fracture bone callus healing status and the load condition that led to the femur plate break are unknown, three load configurations (walking, stumbling, and lateral falling) and four bone reduction factors (50, 20, 10, and 2%) were examined. Comparisons between the Von Mises stress distribution and peaks localization allowed for the identification of the most probable load condition which led to the femur plate breaking. Indeed, the occurrence of a load higher than 6,000 N and characterized by a predominant vertical component causes convergence of the peak stress toward the 11th hole, where the femur plate actually broke. This led to the conclusion that the cause of the event was not a bad design of the osteosynthesis medical device, which has proven to well-perform under the intended use declared by the manufacturer, but a combination of insufficient bone healing and unexpected high load.

## Data Availability Statement

The datasets generated for this study are available on request to the corresponding author.

## Ethics Statement

Written informed consent was obtained from the individual(s) for the publication of any potentially identifiable images or data included in this article.

## Author Contributions

MT, AA, GD, and CB: conceptualization, methodology, and supervision. SN and CB: original draft preparation. All authors contributed to the article and approved the submitted version.

## Conflict of Interest

The authors declare that the research was conducted in the absence of any commercial or financial relationships that could be construed as a potential conflict of interest.
